# Dietary Anthocyanins and Insulin Resistance: When Food Becomes a Medicine

**DOI:** 10.3390/nu9101111

**Published:** 2017-10-12

**Authors:** Tarun Belwal, Seyed Fazel Nabavi, Seyed Mohammad Nabavi, Solomon Habtemariam

**Affiliations:** 1 G.B. Pant Institute of Himalayan Environment and Development, Kosi Katarmal, Almora, Uttarakhand 263643, India; tarungbpihed@gmail.com; 2 Applied Biotechnology Research Center, Baqiyatallah University of Medical Sciences, Tehran P.O. Box 19395-5487, Iran; Nabavisf@gmail.com (S.F.N.); Nabavi208@gmail.com (S.M.N.); 3 Pharmacognosy Research Laboratories & Herbal Analysis Services UK, University of Greenwich, Central Avenue, Charham-Maritime, Kent ME4 4TB, UK

**Keywords:** dietary anthocyanins, insulin resistance, diabetes, obesity

## Abstract

Insulin resistance is an abnormal physiological state that occurs when insulin from pancreatic β-cells is unable to trigger a signal transduction pathway in target organs such as the liver, muscles and adipose tissues. The loss of insulin sensitivity is generally associated with persistent hyperglycemia (diabetes), hyperinsulinemia, fatty acids and/or lipid dysregulation which are often prevalent under obesity conditions. Hence, insulin sensitizers are one class of drugs currently employed to treat diabetes and associated metabolic disorders. A number of natural products that act through multiple mechanisms have also been identified to enhance insulin sensitivity in target organs. One group of such compounds that gained interest in recent years are the dietary anthocyanins. Data from their in vitro, in vivo and clinical studies are scrutinized in this communication to show their potential health benefit through ameliorating insulin resistance. Specific mechanism of action ranging from targeting specific signal transduction receptors/enzymes to the general antioxidant and anti-inflammatory mechanisms of insulin resistance are presented.

## 1. Introduction

The World Health Organization’s (WHO) global obesity data show that, in 2014, more than 1.9 billion adults (18 years and older) were overweight, of which over 600 million were considered obese [1]. These data correspond to the adult population as 39% overweight and 13% obese, while 41 million children under the age of five in the same year were reported as either overweight or obese. This disturbing figure has also been growing with epidemic proportion with obesity number reported to be more than double from 1980 to 2014 [1]. Hand in hand with this fact, the global statistical data for the major obesity associated disease, diabetes, in 2014 were 422 million, while its prevalence has risen from 4.7% in 1980 to 8.5% in 2014 [2]. Today, diabetes is a major cause of disabilities (e.g., blindness and limb amputation), other diseases (e.g., kidney failure and cardiovascular diseases including heart attack and stroke) and death. In the latter case, the WHO estimate for the year 2012 was about 1.5 million deaths directly by diabetes and another 2.2 million deaths related to high blood glucose [2]. 

Diabetes mellitus is a disorder characterized with persistent hyperglycemia in the blood resulting from either deficiency in insulin secretion from pancreatic β cells and/or resistance to insulin. In the case of type-1 diabetes (T1D), the underlying insulin deficiency is a result of pancreatic β-cells destruction by autoimmune-mediated response, while type-2 diabetes (T2D) is mainly caused by insulin resistance, although impaired insulin secretion and β-cell death may also be evident. In both diabetes types, hyperglycemia, if not adequately managed, could lead to significant damage to vital organs but the emphasis of this communication lies on insulin resistance which is prevalent in T2D and/or obesity. 

Being the main source of energy to nearly all cells in our body, glucose concentrations in the blood, its release from complex carbohydrates in the gut, as well as its transport and storage systems are tightly regulated. Central to glucose metabolism is the secretion of insulin by pancreatic β cells following the postprandial excess in the blood, leading to its mobilization and storage in target organs such as the liver, muscles and adipose tissues. The effect of insulin in target organs is regulated through a receptor-mediated signal transduction pathways that has been a great subject of research in the last few decades both for physiological understanding of its action and as targets for novel drugs. The binding of insulin with its membrane receptor triggers autophosphorylation followed by intracellular protein substrates (insulin receptor substrate-1 and -2) phosphorylation. Several upstream and downstream key players in its signal-transduction pathway have been identified including the phosphoinositide 3-kinase (PI3K)/AKT pathway that is known to be involved in the translocation of glucose transporter 4 (GLUT-4) from intracellular vesicles to the cell membrane [3,4]. Since GLUT-4 is involved in glucose transport in muscles and adipose tissues, it is a major therapeutic target for T2D [5]. As explained in the following sections, the mitogen-activated protein kinase (MAPK), adenosine monophosphate-activated protein kinase (AMPK) and the stress-activated c-Jun *N*-terminal kinase (JNK) pathways are other important signaling key players in insulin action that may be targeted by potential therapeutic agents [6,7].

If the insulin released from pancreatic β-cell failed to adequately bind or trigger the signal transduction pathway that leads to its expected physiological response, a condition known as insulin resistance is developed. A decrease in insulin effectiveness means that more and more insulin release is required to achieve the normal control of glucose and other (e.g., lipids) metabolisms. Interestingly, persistent hyperglycemia (diabetes), hyperinsulinemia, fatty acids dysregulation (e.g., hyperlipidemia) can also lead to insulin resistance [4,8]. In this context, this review is addressing the potential insulin resistance modulatory effect of dietary natural products collectively called anthocyanins. Furthermore, this approach is clinically relevant, as drugs such as rosiglitazone and pioglitazone are used to treat diabetes and/or insulin resistance through their agonistic effect on peroxisome proliferator activated receptor-gamma (PPAR-γ) [9]. Many natural products that act through similar mechanism have also been identified [10]. Considering the various side effects of the clinically useful anti-diabetic drugs, the search for novel anti-diabetic agents from natural sources including multifunctional flavonoids is currently gaining a lot of attention [11,12,13,14].

## 2. Chemical Diversity of Dietary Anthocyanins

**Figure 1:**
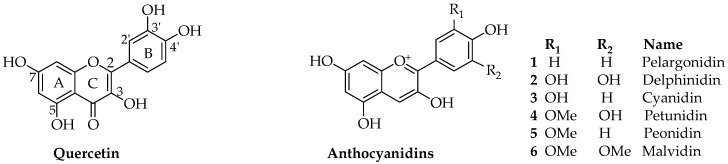
The common flavonoid skeleton as exemplified by quercetin and the anthcyandins.

**Figure 2:**
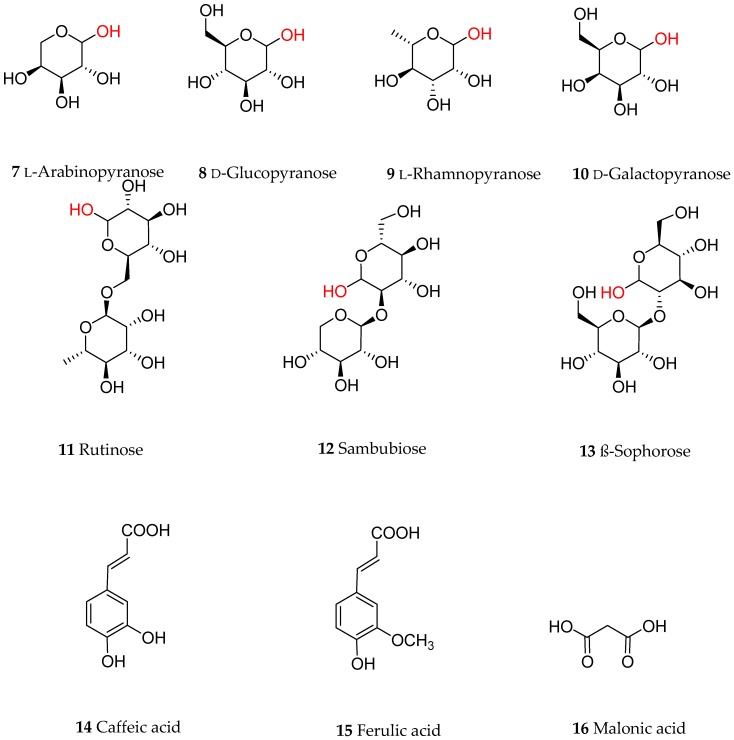
Common sugars and ester derivatives of anthocyanins discussed in this article. The linking position of the sugars with the flavonoid skeleton is shown in red.

Anthocyanins are a group of polyphenolic natural products that belong to a broad class of secondary metabolites collectively called flavonoids. Structurally, flavonoids, composed of a 15-carbon skeleton, derive from two distinct biosynthetic pathways: the shikimic acid pathway giving rise to the C6-C3 cinamate derivatives and the C6 acetate derived precursors. The general structural feature of flavonoids is shown in Figure 1 and characterized by two aromatic rings (ring A and B) joined together by a three linking carbon chain that may form a third cyclic structure normally designated as ring C. The most common structural diversity of flavonoids arises due to the presence or absence of the 4-ketone functional group, C2-C3-double bond, oxygenation at C-3, attachment site of the ring-B at the linking three-carbon (C-2, C-3 or C-4 positions), etc. The further source of flavonoids’ diversity is the number of hydroxyl substitutes at the two aromatic rings (A and B) and further *O*- or *C*-glycosylation and *O*-acetylation/etherification. Anthocyanins exhibit extended conjugated double bonds in all three rings, resulting from the flavylium ion or 2-phenylbenzopyrilium that give rise to their intense color pigments (Figure 1). Anthocyanins do also possess sugar units attached to the flavonoid skeleton, normally through *O*-linkage, and hence are water soluble. The flavonoid or aglycone unit of anthocyanins is called the anthocyanidins, which may also be present in plants in unglycosylated forms. The most common anthocyanidins encountered in plants are the pelargonidin, cyanidin, peonidin, delphinidin, petunidin and malvidin (Figure 1). Depending on the number of hydroxyl groups, site of attachment of the sugar unit(s), type of sugar (e.g., arabinose, galactose, glucose, rhamnose, and xylose frequently encountered), glycosidic linkage (α or β linkage) and complexity of the sugars (mono-, di- and trisaccharide) diverse group of anthocyanins are known to occur in plants. Most of the bioactive anthocyanins discussed in the following sections possess one sugar unit mostly as 3-*O*-glycoside forms of arabinose (**7**), glucose (**8**), rhamnose (**9**) and galactose (**10**) in their pyranoside forms (Figure 2). Diglycoside forms including rutinose (**11**), sambubiose (**12**), and sophorose (**13**) are also common, while acylation with caffeic (**14**), ferulic (**15**) and malonic (**16**) acids (Figure 2) are presented in the structural diversity of the bioactive anthocyanins (Table 1).

## 3. Natural Occurrence of Dietary Anthocyanins

Being bright in coloration, ranging red, pink, purple and blue, anthocyanins are the principal components of pigmented plant parts such as flowers and fruits. Their functions to the plant that produce them include aiding pollination [15,16] and seed dispersal by attracting insects and other animals, while their antioxidant effects have been implicated to the plants survival, especially in UV prevalent high altitude environments [17]. Besides the different hydroxyl substitutions of the anthocyanidins skeleton giving rise to different colorations, anthocyanins could undergo chemical transformation depending on various pH conditions to give rise to variable colors. Understanding this natural properties of anthocyanins is thus important in maintaining their natural color and stability during industrial processing, storage and shelf-life of anthocyanin-rich foods and/or colorants [18,19]. This remarkable chemical diversity is generally manifested in the anthocyanin-rich fruits and vegetables. The recent interest in the blue, red and purple colored cereal products such as purple corn and red and black rice grains are also due to their anthocyanin content that are claimed to have a plethora of health benefits. The most common sources of dietary anthocyanins remain berries, including blackberries, bilberries, chokeberries, elderberries, cranberries and raspberries. Many other highly colored fruits such as black currant, cherry, grape, strawberry, colored cabbage, eggplant and radish are also known to have high levels of anthocyanins. Some of these products such as the berries and grapes are fermented to yield beverages that are also rich sources of anthocyanins. In the following sections, some of these plants as a source of anthocyanins are scrutinized for their potential insulin sensitizing effects in mammalian cells. 

## 4. Dietary Anthocyanins and Insulin Sensitivity/Resistance

### 4.1. In Vitro Protective Activity: Insulin Resistance Diabetic/Obese Condition

A summary of few selected key findings showing the enzyme inhibitory activities and various other effects in in vitro cell culture-based experiments are shown in Table 2. α-Glucosidase and pancreatic α-amylase are the major targets of anthocyanins that undoubtedly contribute to their anti-diabetic effects [20,21,22,23,24,25,26,27,28,29,30,31,32,33,34,35,36,37,38]. This means that anthocyanins share some common anti-diabetic mechanism with drugs such as acarbose that target carbohydrate digestion in the gut thereby limiting the availability of glucose released to the blood. The research findings so far appear to suggest that anthocyanins such of the common cyanidin, plegonidin, delphinidin and petunidin glycosides are effective carbohydrate digestive enzyme inhibitors. In addition, in vitro studies on cell culture including in insulin resistance hepatocytes (HepG2 cells), human adipocytes such as the classical 3T3-L1 adipocytes, the rat liver cells including H4IIE cells, muscle cells such as L6 myotubes, rodent pancreatic β-cells and satellite cells have been conducted to evaluate the potential of anthocyanins in insulin resistance [39,40,41,42,43,44,45,46,47,48] (Table 2). It can be summarized from these reports that anthocyanins may increase insulin sensitivity and glucose uptake in vital organs such as the muscles and adipose tissues and hence can ameliorate insulin resistance under diabetic condition. In addition, anthocyanins exert positive effect on adipocytes cell culture by suppressing lipogenic factors [42,43]. 

A few studies on the structure–activity relationship (SAR) of anthocyanins with respect to their anti-diabetic potential have been conducted. For instance, the intestinal α-glucosidase and pancreatic α-amylase inhibition activity was found higher in cyanadin 3-*O* position substituted with glucose or galactose. Among the entire tested cyanadin analog, the higher activity was recorded in cyanidin-3-galactoside followed by cyanidin-3-glucoside, cyanidin and cyanidin-3,5-di-*O*-glucoside [23]. It is interesting to see that C-3-*O*-substitution with glucose increases the activity, while one more substitution of glucose at C-5-*O*-position decreases the activity. In another SAR activity study, acylation of anthocyanins with caffeic or ferulic acid was found to enhance α-glucosidase inhibitory effect [20]. On the other hand, methylation and methoxylation drop the activity while the glucose substitution at 3-*O*-position increases the α-glucosidase inhibition. Similar results were reported from other studies with a higher α-glucosidase inhibition activity of cyanidin-3-sambubioside followed by cyanidin-3-glucoside and cyanidin [22]. In another study, fifteen anthocyanins were isolated from *Cornus* fruits and tested for insulin secretion in rodent pancreatic β-cells. Among all the anthocyanins, the higher activity was recorded for cyanidin-3-glucoside and delphinidin-3-*O*-glucoside as compared to their galactoside or sambubioside substitution and thus with increasing number of hydroxyl groups in anthocyanin B ring, insulin secretion also increases [48]. In addition, when rat adipocytes were tested with anthocyanins-cyanidin-3-*O*-glucoside and cyanidin, a total of 633 genes were upregulated under cyanidin-3-glucoside treatment as compared to 427 under cyanidin, which showed structural differences attributes to the expression of different genes and thus leads to variable functional responses [49,50].

### 4.2. In Vivo Protective Activity: Insulin Resistance Diabetic/Obese Condition

**Figure 3:**
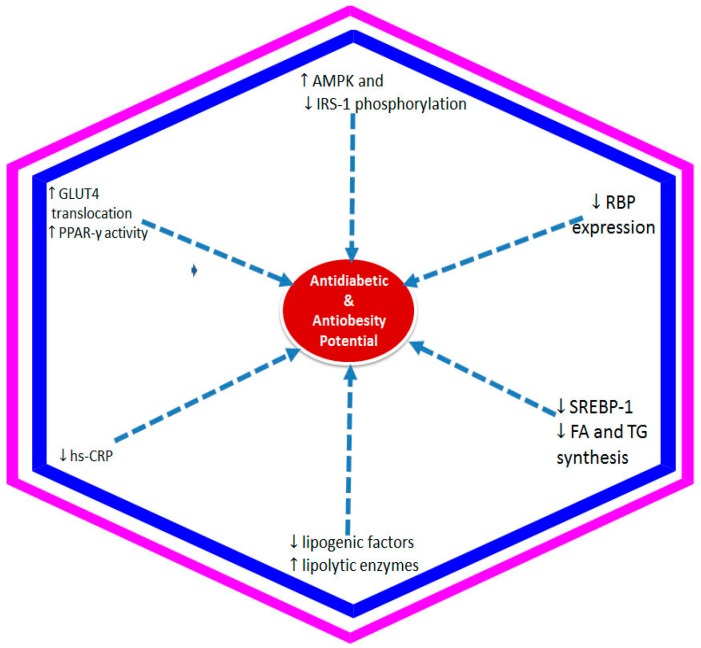
Underlying mechanism of anthocyanins against insulin resistance associated with diabetes and/or obesity. The decrease in insulin resistance and enhancement of insulin sensitivity by anthocyanins in target organs have been shown to be mediated through activation of the AMPK (adenosine monophosphate-activated protein kinase) and downregulated the serine phosphorylation of IRS-1 (insulin receptor substrate 1), enhanced GLUT4 (glucose transporter 4) translocation by increasing the activity of PPAR-γ (peroxisome proliferator activated receptor-gamma), lowering the hs-CRP (high sensitivity C reactive protein) concentration, and reduction of retinol binding 4 (RBP4) expression. The reduction of weight gain by anthocyanins is also reported through mechanisms including reduction in the SREBP-1(sterol regulatory element-binding protein 1) mRNA level and inhibition of fatty acid (FA) and triglycerol (TG) synthesis enzymes as well as downregulation of lipogenic factors and upregulation of lipolytic enzymes.

A vast numbers of in vivo protective activities of anthocyanins against insulin resistance diabetic and obesity condition have been performed on insulin resistant diabetic obese animal model using either anthocyanin rich extract or isolated compounds (Table 3). These include extracts from fruits/berries, such as *Cornus mas*, *Ficus benghalensis*, mulberry, blueberry bilberry, *Morus*, grape, *Aronia* berry, sweet orange, sweet cherry, etc. The isolated anthocyanin compounds such as cyanidin, delphinidin and pelorgonidin glucosides were also tested for their in vivo activity. These compounds along with anthocyanin rich extracts were found to be effective in ameliorating the insulin resistance condition and also increase insulin sensitivity, decrease body weight gain and accumulation of lipids [40,51,52,53,54,55,56,57,58,59,60,61,62,63,64,65,66,67,68,69,70,71,72,73,74,75,76,77,78,79,80,81,82,83,84,85,86,87,88,89,90,91]. Other than fruits/berries, anthocyanins extracted from purple corn, black rice and black soybean have also been tested in vivo against insulin resistance diabetic and obesity conditions and found to be effective (Table 2). These data along with the in vitro evidence presented in Table 2 suggest different mechanisms being responsible for the protective activity of these anthocyanins (Figure 3). The most relevant mechanisms postulated so far include, increasing GLUT-4 translocations, activation of the AMPK and lipolytic enzymes, decreasing the serine phosphorylation of IRS-1 (insulin receptor substrate 1), downregulating retinol binding 4 expression, SREBP-1 (sterol regulatory element-binding protein 1) mRNA level and inhibition of fatty acid and triglycerol synthesis enzyme and lypogenic activity. These all played effective role in increasing insulin sensitivity and reverse diabetic/obese condition (e.g., [41,63,67]).

## 5. Clinical Study

A few clinical studies have been conducted on the effect of anthocyanins against insulin resistance under diabetes and/or obesity conditions. In one study, for example, anthocyanins from bilberry (*Vaccinium myrtillus*) and black current (*Ribes nigrum*) was tested in patients with type-2 diabetic condition and shown to significantly decrease the serum LDL (low-density lipoprotein) cholesterol by 7.9%, triglycerides by 23.0% and increased HDL (high-density lipoprotein) cholesterol by 19.4% [92]. In addition, it lowered fasting plasma glucose by 8.5% and in homeostasis model assessment for insulin resistance by 13% [93]. In another study, smoothie containing 22.5 g blueberry powder (50:50 mixture of *Vaccinium ashei* and *Vaccinium corymbosum*) was taken twice daily for 6 weeks by obese and insulin resistance patients. A 67% of patient showed an increased insulin sensitivity of at least 10% or greater as compared to control [93]. A meta-analysis was conducted on 1997 female participants of age between 18 and 76 years on consumption of flavonoids and its subclass (flavanones, anthocyanins, flavon-3-ols, polymeric flavonoids, flavanols, and flavones) was also calculated from food frequency questionnaires using USDA (U.S. Department of Agriculture) database. A significant lowering effect on peripheral insulin resistance was seen in women consuming higher anthocyanins and flavones-rich food [94]. 

A pilot study with 22 diabetic patients (14 women and 8 men) were employed by Esmaillzadeh [95] to assess the potential cholesterol-lowering effect of patients consuming concentrated pomegranate 40 g/day over a period of eight weeks. At the end of the eighth week, it was reported that a significant reductions in total cholesterol and LDL-cholesterol without any change in the serum triacylglycerol concentrations. In a similar experiment, Rashidi et al., [96] studied the effect of daily consumption of 45 g concentrated pomegranate for 3 month in diabetic patients. Even though the cholesterol and LDL concentrations on the treatment group was lower, a significant effect was not observed in what appears to be a contradiction with the other studies (e.g., [95]). 

Edirisinghe et al. [97] recruited 24 overweight adults to study effect of strawberry consumption on high-carbohydrate, moderate-fat meal diet. In their placebo beverage controlled cross-over study, they have shown that strawberry beverage could attenuate the postprandial inflammatory response as demonstrated from the lower level of high-sensitivity C-reactive protein and interleukin-6 (IL-6) in the treatment group. The increased postprandial prevalence of pelargonidin sulfate and pelargonidin-3-*O*-glucoside following consumption of strawberry also suggest the potential role of anthocyanins in the observed biological activity. A further example of a small pilot study is on açaí palm (*Euterpe oleracea* Mart.) where 10 overweight adults (BMI ≥ 25 kg/m^2^ and ≤ 30 kg/m^2^) took 100 g açai pulp twice daily for 1 month [98]. The suppressive effect of this treatment on the postprandial increase in plasma glucose following the standardized meal was reported [98].

Unfortunately, clinical studies demonstrating the true potential of anthocyanin supplementation are far from complete. Those studied so far are carried out in a small number of subjects with no clear standardization formula applied to the drug preparations. Beyond inconsistencies in the preparations of the plant materials, dosing regimens and other clinical study parameters such as patient groups, blinding, etc. are other issues that all need to be addressed in the future. In this connection, a growing number of clinical trials on anthocyanins are being conducted (Table 4) and perhaps the discrepancies between the tremendous potential benefit of these class of compounds in ameliorating insulin resistance and tackling T2D/obesity observed in vitro and in vivo and that in humans will be resolved in the very near future. A lot more work is therefore required to further validate the true clinical potential of anthocyanins if we have to consider them as drugs—or potential benefits far more than medicinal foods (see the general discussion section below). 

## 6. General Summary and Conclusions 

There appear to be overwhelming in vitro and in vivo, and few clinical studies data to suggest that dietary anthocyanins could ameliorate insulin resistance and offer health benefits in diabetic conditions. One of the key features of their pharmacological effects appear to be linked to multiple mechanisms ranging from inhibiting carbohydrate digestion in the gut, pancreatic β-cell protection and insulin secretion to enhancing insulin sensitivity in vital organs. As insulin resistance is closely linked to obesity, some of the common mechanisms for anthocyanins effect on insulin resistance is summarized below.

Inflammation has been established to be the best characterized link between obesity and insulin resistance. In fact, obesity is regarded as a state of low-grade inflammation where pro-inflammatory cytokines and chemokines are continually released by adipocytes and immune cells leading to the recruitment and infiltration of macrophages and other leucocytes population. The upregulation of inflammatory cytokines (mainly tumour necrosis factor (TNF)-α and IL-6) and their role in insulin resistance has been established and in this connection, readers are directed to the various review articles on the subject [99,100,101,102]. Through activation of the NF-κB pathway, these pro-inflammatory cytokines suppress the insulin signal transduction pathway including the PI3K-AKT pathway (also known as the protein kinase B (PKB)) through which insulin mediates glucose uptake while gluconeogenesis is inhibited. Likewise, an increase in the level of triglycerides is associated with insulin resistance through the same mechanism. The other closely related insulin signaling pathway is the MAPK pathway which together with the PI3K-AKT pathway initiate gene expression, cell growth and differentiation. Agents that promote the phosphorylation of IRS1 (e.g., the serine kinases that phosphorylate serine 307) such as the NF-κB and C-jun N-terminal kinase 1 (Jnk1) in the JNK/AP-1 pathway could diminish the insulin response. Hence, in addition to the NF-κB through action on the IK-κB, cytokines and fatty triglycerides that activate the ser/thr kinases such as Jun NH2-terminal kinase (JNK) and protein kinase C (PKC) pathways suppress insulin signaling [100,101]. The known anti-inflammatory effect of anthocyanins is therefore expected to play major role in their potential benefit in ameliorating insulin resistance. In vivo experiments, for example, have shown that anthocyanins such as cyanidin-3-*O*-β-glucoside can suppress monocyte infiltration [103] and have potential to treat lung inflammation [104] as well as atherosclerosis-related diseases. At the molecular level, their mechanisms of anti-inflammatory effect include inhibition of cyclooxygenase [105] and the MAPK and NF-κB signaling pathways [106]. Through the combined effect on reactive oxygen species (ROS) and NF-κB, the inhibitory effect of anthocyanins and/or anthocyanidins have been reported [107], while other reports indicate direct inhibitory effect on the expression of inflammatory genes [108]. Other studies also revealed that anthocyanins (e.g., Mulberry anthocyanin extract) ameliorate insulin resistance in vitro by regulating the PI3K/AKT pathway [109]. 

Along with inflammation, the role of oxidative stress in obesity and insulin resistance has been the subject of intense debate in recent years. The growing body of evidence now suggests that ROS generation under the state of obesity is upregulated while antioxidant defenses diminish over time and this trend is even greater after the onset of diabetes. Hence, 3T3-L1 adipocytes lose their sensitivity to insulin in vitro when exposed to H_2_O_2_ even in micromolar concentration range [110]; while insulin resistance in this cells induced by TNF-α could be reversed by boosting antioxidant defenses [111] (e.g., β-carotene accumulation) suggesting the link between oxidative stress and insulin resistance. Similarly, other natural products including fermented rice bran extract [112] and green tea flavonoids (catechins) [113] have been shown to improve insulin resistance through antioxidant mechanisms. Undoubtedly, one of by far the most common mechanisms involved in the health benefit of anthocyanins including in insulin resistance is therefore related to their proven antioxidant effects. As with other flavonoids, the phenolics nature of these compound accounts to the antioxidant effects but their optimal structure of the catechol functional group is the key determinant that we have shown to play pivotal role for the antioxidant potential of natural products [14,114,115,116,117,118,119,120,121,122,123,124,125,126,127]. The numerous hydroxyl positions of the flavonoid skeleton coupled with the fully extended double bonds including in the C-ring play important in the free radicals and/or ROS scavenging properties of anthocyanins. Accordingly, cyanidin-3-*O*-β-d-glucoside and related anthocyanins have been demonstrated to protect macromolecules including DNA from oxidative damage [128,129,130] or cellular damage induced in vitro by ROS [131]. Organoprotective effects such as the heart in the ischemia-reperfusion injury [132] have also been shown for anthocyanins. In addition to direct scavenging effect, the generation of ROS induced by various agents has been shown to be suppressed by anthocyanins including cyanidin-3-*O*-β-glucopyranoside [133]. As expected, the aglycones of anthocyanins also possess antioxidant effects as well as protective ability in animal cells against oxidative injury and/or cell death [134,135]. Anthocyanins have also been shown to induce phase II enzymes through the antioxidant response element pathway [136]. Animal models of diabetes also revealed that anthocyanins (boysenberry anthocyanins) inhibit oxidative stress by increasing the level of glutathione [137], while other similar studies have shown an increase in the level of antioxidant enzymes such as catalase, superoxide dismutase (SOD), and glutathione peroxidase (GPx) [138]. Hence, the overall antioxidant effects of anthocyanins could be mediated both through direct effect on ROS generation and/or scavenging and enhancement of antioxidant defenses. Considering the deletion of pancreatic β-cells involve oxidative mechanism and the later stage of diabetes complications such as the glucose oxidation-induced damage and cardiovascular problems are closely linked to oxidative stress, antioxidant mechanism of anthocyanins could offer a lot more health benefits than just enhancing inulin sensitivity. In this regard, the direct β-cell protective effects of anthocyanins, such as those from blueberries and cyanidin-3-*O*-glucoside isolated from mulberry fruit, have been well documented [92,139]. On the other hand, the hyperglycemia-induced formation of advanced glycation end products that is correlated to oxidative stress associated with diabetic complication has to be overcome through antioxidant therapy. The demonstration of dietary anthocyanins in diabetes treatment as evidenced from various studies including anthocyanin-rich extract from black rice [56] and numerous others highlighted in Table 2 and Table 3 are classical example of potential dietary intervention of insulin resistance by these group of natural products. 

One of the well-established mechanism of anti-diabetic drugs is through upregulation of expression of the glucose transporter GLUT-4 that anthocyanins have been shown to be effective. For example, the amelioration of insulin resistance and anti-diabetic effects in the streptozotocin-induced diabetic rat model by anthocyanins has been shown to be coupled with GLUT-4 regulation [59]. The AMPK pathway has also been emerged as a major drug target for diabetes and related diseases given its crucial regulatory role in energy metabolism involving glucose and lipids [140]. The increased phosphorylation of the AMPK pathway by anthocyanin such as those from mulberry fruit extract could not only increase glucose uptake but also inhibit gluconeogenesis and stimulates glycogen synthesis [141]. The improvement of glucose homeostasis in diabetic mice by cyanidin-3-*O*-β-glucoside [142] and dietary anthocyanin-rich bilberry extract [67] has been shown to be mediated through activation of the AMPK pathway. The study by Huang et al. [143] on the anti-diabetic effect of purple corn extract on C57BL/KsJ db/db mice model also shed some light on the involvement of the AMPK pathway in the anti-diabetic potential of anthocyanins. The study revealed that purple corn extract increased the phosphorylation of AMPK and decreased phosphoenolpyruvate carboxykinase, glucose 6-phosphatase genes in liver, while the GLUT4 expressions in skeletal muscle was augmented. These activities were coupled with anti-diabetic effect as assessed by reduction in the fasting glucose level and HbA1c levels. Hence, the interlinking role of oxidative stress and the AMPK pathway in glucose metabolism and diabetes control by anthocyanins is a proven productive area of interest that gained momentum in recent years [41,144].

Another mechanism for anthocyanin’s health benefit is through effects on the various functional aspects of adipocytes that is linked to insulin resistance. Anthocyanin extracts from black soybeans, which were shown to be composed of cyanidine-3-*O*-glucoside (68.3%), delphinidin-3-*O*-glucoside (25.2%), and petunidin-3-*O*-glucoside (6.5%), not only reduced lipid accumulation in vitro but also suppressed the expression of the PPAR-γ [145]. Given that the thiazolidinediones (TZD) classes of anti-diabetic drugs are effectively used due to they being the PPAR ligands through which they initiate adipocytes genes activation and cellular differentiation, the effect of anthocyanins on this system is another interesting dimension of diverse mechanisms. Anthocyanins could also lower the level of circulating free fatty acids through direct effect on lipolysis in adipose tissue. In this regard, cyanidin-3-*O*-β-glucoside has been shown to suppress the expression of adipose triglyceride lipase in cultured 3T3-L1 adipocytes while at the same time increasing the activity of the AMPK [146]. One more adipocyte regulator that gained interest in recent years has been the retinol binding protein 4 (RBP4) which by its own right can be classified as an adipocytokine. The level of RBP4 in the blood and adipose tissue appears to be increased in obesity and/or diabetes. The correlation between this dysregulation and development of insulin resistance has been reviewed (e.g., [147,148,149]). Interestingly, anthocyanin (e.g., cyanidin-3-*O*-glucoside) have been shown to downregulate the RBP4 in the white adipose tissue in type 2 diabetic mice while at the same time upregulating the GLUT-4 and suppressed adipocytokines (monocyte chemoattractant protein-1 and tumor necrosis factor-α) [86]. 

Given the diverse mechanism of action of anthocyanins that is implicated in tackling various disease conditions, their absorption and pharmacokinetic profile have also been scrutinized in recent years. Anthocyanins appear to be absorbed throughout the gut including in the stomach (e.g., [150]) and the colon [151]. Being glycosides, they appear to exploit the glucose transport systems (GLUT-1/2) in the small intestine and extensive research articles describing the detailed mechanisms have been published [152,153,154,155]. These studies suggest that intact anthocyanins are absorbed from the ilium and reach to vital organs such as the liver, blood, kidney and ocular tissues while other derivatives such as the common glucoronate and methylated products are also common [156,157,158]. Anthocyanins can also be converted by the gut microbiota into other smaller products such as aromatic acids, which could also contribute to their known pharmacological effects (e.g., [159,160,161,162]). 

In conclusion, dietary anthocyanins appear to be targeting insulin sensitivity through diverse mechanisms and have potential to modulate disease states like diabetes. Their modulatory effect in insulin resistance appears to be mediated via targeting the various specific insulin signal transduction pathways of enzymes/receptors and also through general antioxidant and anti-inflammatory mechanisms. With respect to them being used as medicine by their own right, comprehensive clinical studies with standardized anthocyanins components, doses, blinding and large number of subjects need to be performed to ascertain their true therapeutic potential for treating diabetes and associated diseases. In the meantime, their dietary benefits appear to be extended to enhancing insulin sensitivity that is often linked to obesity and the development of diabetes. In this regard, their multifunctional nature, expressed in our title, when food become a medicine, is a well-deserved statement that begs for further studies on these promising natural products. 

## Figures and Tables

**Table 1:** Structural diversity of anthocyanins with reported ameliorative effect on insulin resistance *.

Compound	Cyanidin Glycosides	Compound	Delphinidin, Pelargonidin and Peonidin Glycosides
**17**	Cyanidin-3-*O*-arabinoside	**26**	Delphinidin-3-*O*-galactoside
**18**	Cyanidin-3-*O*-galactoside	**27**	Delphinidin-3-*O*-glucoside
**19**	Cyanidin-3-*O*-glucoside	**28**	Delphinidin-3-*O*-sambubioside-5-*O*-glucoside
**20**	Cyanidin-3-*O*-glucosyl-rutinoside	**29**	Pelargonidin-3-*O*-galactoside
**21**	Cyanidin-3-*O*-rutinoside	**30**	Pelargonidin-3-*O*-glucoside3
**22**	Cyanidin-3-*O*-sambubioside	**31**	Pelargonidin-3-*O*-rutinoside
**23**	Cyanidin-3-*O*-sophoroside	**32**	Pelargonidin-3-*O*-(2-*O*-(6-*O*-(E-3-*O*-(β-d-glucopyranosyl)caffeyl)-β-d-glucopyranosyl)-6-*O*-*E*-caffeoyl-β-d-glucopyranoside)-5-*O*-β-d-glucopyranoside
**24**	Cyanidin-3,5-*O*-diglucoside	**33**	Peonidin-3-*O*-rutinoside
**25**	Cyanidin-3-*O*-malonylglucoside	**34**	Peonidin-3-*O*-(2-*O*-(6-*O*-*E*-feruloyl-*β*-d-glucopyranosyl)-6-*O*-*E*-caffeoyl-β-d-glucopyranoside)-5-*O*-β-d-glucopyranoside

* The effect of these compounds on insulin resistance is summarized in Table 2 and Table 3.

**Table 2:** In vitro modulatory effects of anthocyanins against insulin resistance and diabetes.

Anthocyanins	Plant Name and Part Used	In Vitro Model/Activity	References
Purified acylated anthocyanins: e.g., **32**	*Morning glory* (red flower)	α-Glucosidase/Enzyme inhibition	[20]
6-*O*-Caffeoylsophorose of the diacylated anthocyanin, **34**	Fermented purple-fleshed sweet potato	α-Glucosidase/Enzyme inhibition	[21]
Purified acylated anthocyanin: **22** but not **19** or cyanidin (**3**)	*Viburnum dilatatum* fruits	α-Glucosidase/Enzyme inhibition	[22]
Anthocyanins **18**, **19** and **24**	Synthetic source	α-Glucosidase and pancreatic α-amylase/Enzyme inhibition	[23]
Methanolic extracts and purified anthocyanins: **24** as well as cyanidin (**3**)	Noble Muscadine grape - whole fruit and skin	α-Glucosidase and pancreatic lipase/Enzyme inhibition	[24]
Anthocyanins enriched water extract	*Ipomoea batatas* (purple sweet potato)	α-Amylase and α-glucosidase/Enzyme inhibition	[25]
Aqueous ethanol (70%) extract containing **19**, **21**, **31** and **33**	Pulp of Sweet cherry cultivars	α-Glucosidase/Enzyme inhibition	[26]
Ethanol extract	*Syzygium dessiflorum* fruits	α-Amylase/Enzyme inhibition	[27]
Smoothies containing anthocyanins **19**–**21**, **23** and **33**	Fruits of sour cherry (*Prunus cerasus, Prunus persica, Prunus armeniaca* and plum)	α-Amylase and α-glucosidase/Enzyme inhibition	[28]
Aqueous extract	Roselle (*Hibiscus sabdarilfa)* flowers	α-Amylase and α-glucosidase/Enzyme inhibition	[29]
Methanol extract	*Aegle marmelos* fruit pulp	α-Amylase and α-glucosidase/Enzyme inhibition; L6 rat skeletal muscle cells/Increase glucose uptake	[30]
Aqueous and methanol extracts	Strawberries fruits	α-Amylase and α-glucosidase/Enzyme inhibition	[31]
Aqueous extract	Brazilian strawberry cultivar *(Fragaria x ananassa)*	α-Amylase and α-glucosidase/Enzyme inhibition	[32]
Aqueous extracts	Red current, black current, red and green goose berries	α-Amylase and α-glucosidase/Enzyme inhibition	[33]
Aqueous, methanolic, and acetic acid extracts; Major component are **17**–**19**	black chokeberry (*Aronia melanocarpa)* berries	α-Amylase and lipase/Enzyme inhibition	[34]
Aqueous and ethyl acetate extracts	Serviceberry plant samples (leaves, twig and berries)	α-Glucosidase/Enzyme inhibition	[35]
Purified flavonoids including anthocyanins	Standard chemicals	α-Glucosidase/Enzyme inhibition	[36]
Aqueous methanol extracts containing anthocyanins (**21**, **24** and **31**)	Fig (*Ficus carica)* fruits	α-Amylase and α-glucosidase/Enzyme inhibition	[37]
Ethanol and methanol extracts and purified anthocyanins: **19** and **22**	Elderberries (*Sambucus nigra*)	α-Glucosidase and α-amylase/Enzyme inhibition; skeletal muscle cells/Stimulate glucose uptake	[38]
Anthocyanin-rich formulation and purified anthocyanin: **28**	Maqui berry (*Aristotelia chilensis*)	H4IIE rat liver cells and L6 mycotubes/Decrease glucose production, increase glucose uptake and enhanced insulin stimulated downregulation of gluconeogenic enzyme, glucose-6-phosphatase	[39]
Aqueos extract containing **19** and **25**	Rutgers Scarlet Lettuce	H4IIE rat hepatoma cells/Inhibition of glucose production	[40]
Acidified ethanol extract and purified anthocyanins: **19**, **21** and **30**	Mulberry (*Morus alba*)	HepG2 cells/Increase glucose uptake	[41]
Purified anthocyanin: **19**	Standard reference	Human omental adipocytes and 3T3-L1 cells/Increase glucose transport, GLUT4 membrane translocation and insulin sensitivity (insulin like activity);	[42]
Aqueous extract containing high concentration of acylated cyanidin and peonidin	Purple sweet potato	3T3-L1 adipocytes/Suppress leptin secretion and expression of lipogenic and inflammatory factors; promoted lipolytic action	[43]
Standardized extract containing 25% anthocyanins	Bilberry	3T3-L1 cell line/Decrease adipocyte differentiation via insulin signaling pathway	[44]
Fermented Juice	Lowbush blueberry fruits	Insulin sensitive cultured muscle cells and adipocytes/Stimulate glucose uptake; increase insulin sensitivity	[45]
Ethanol extract	Canadian lowbush blueberry (*Vaccinium angustifolium)* fruits	Replicating βTC-tet cells/Increase proliferation	[46]
Aqueous extract	*Eugenia jambolana* seeds and pulp	Cultured Islets of Langerhans cells of normal and diabetic rats/Stimulate insulin release	[47]
Purified anthocyanins: **18**, **19**, **27** and **29**	Cornus fruits (*C. officinalis* and *C. mas*)	Rodent pancreatic β-cells (INS-1832/13)/Increased insulin secretion and prevent insulin resistance	[48]

**Table 3:** In vivo effects of anthocyanins against insulin resistance or diabetes/obesity.

Anthocyanins *	Plant Name and Part Used	Animal Model	Anti-diabetic and/or Anti-obesity Activity	References
Juice	Acerola (*Malpighia emarginata* DC.) fruits	Diabetic Wister rats	Reduction in blood glucose level, cholesterol and triglyceride	[51]
Juice	*Aronia melanocarpa* Fruits	Diabetic rat	Lower glucose and lipid level	[52]
Juice	*Aronia melanocarpa* (black chokeberry)	Obese mice under high fat diet	Lower lipid level	[53]
Athocyanin fractyion of ethanol extract	*Berberis integerrima* fruits	STZ-induced diabetic rats	Reduce glucose level and increase glycogen	[54]
Juice containing **19** and **25**	Blackberries (*Rubus adenotrichos* Schltdl. Cv. “vino”*)*	STZ-induced diabetic rats	Decreases the levels of glucose, triacylglycerols and cholesterol	[55]
Anthocyanin rich fractions	Black rice (*Oryza sativa*)	Rats fed high fructose rich diet for 4 weeks	Increase plasma insulin level and insulin sensitivity; prevent insulin resistance; hypolipidemic effect	[56]
Anthocyanin rich preparation	Black soybean seed coat	STZ induced diabetic rats	Protect pancreatic tissue from apoptosis, regulation of glucose transport; prevent insulin resistance; hypolipidemic effect	[57]
Anthocyanin rich preparation	Black soybean (*Glycine max* L.)	Rats fed with high fat diet	Decrease body weight gain; suppress weight gain in liver, epidymal and perirenal fat pads; improve lipid profile, serum triglyceride and cholesterol level	[58]
Juice	Blueberries	Obese rodent fed with high fat diet	Improve insulin resistance and glucose tolerance	[59,60,61]
Powder diet	Blueberries	Obese and insulin resistance mice fed with 60% high fat diet and 4% blueberries	Lower plasma glucose; increase insulin sensitivity; reduce adipocyte cell death	[62]
Juice/Powder	Blueberries	Obese rodent	Decrease body weight gain and lipid accumulation; increase insulin sensitivity	[59,63,64]
Powder	Blueberries	Obese rat	Anti-obesity effect; increase glucose absorption	[65]
Powder	Blueberry	Obese mice fed with high fat diet	Decrease body weight and body fat accumulation	[66]
Powder	Blueberry	Obesity prone rat (Zucher fatty and Zucker lean)	Reduces triglycerides, fasting insulin; improve insulin sensitivity	[60]
Extract (unknown)	Blueberries (*Vaccinium myrtillus*) or Bilberries (*Vaccinium cyanococcus*)	Type-2 diabetic male KK-Aγ mice	Amloriate insulin sensitivity; improve diabetic condition; suppress glucose production and lipid content in the liver	[67]
Methanol extract and anthocyanin fraction	Blueberry (*Vaccinium angustifolium*)	Diabetic mice	Hypoglycemic activity	[68]
Powdered formulation or juice	Blueberry, Black current, Concord grape, Black raspberry and Maqui berry	Obese mice with high fat diet	Improve insulin sensitivity	[69]
Fermented beverage	Blueberry and blackberry	Obese mice fed with high fat diet	Reduce fasting blood glucose level; prevent obesity	[70]
Juice	Blueberry and Mulberry	Obese mice fed with high fat diet	Decrease body weight gain and serum cholesterol level; reduce insulin resistance, lipid accumulation and leptin secretion	[64]
Powder	*Blueberry (Vaccinium ashei)*	Obese male mice under high fat diet	Decrease serum glucose; improve lipid profile	[71]
Powder	Blueberry	Female mice fed with high fat diet	Supplement prevent glucose and insulin tolerance in obese post-menopausal mice	[61]
Spraydried (CellBerry^®^)	Chokeberry	Rats fed with high fructose-rich diet	Reduce weight gain; modulate insulin, adipogenic and inflammatory signaling pathways	[72]
Purified anthocyanins: **18**, **29** and **26**	Cornelian cherry (*Cornus mas*)	Obese and insulin resistance mice fed with high fat diet	Decrease body weight and accumulation of lipids and triglyceride in the liver; increase insulin level; preserve islet architecture	[73]
Ethanol extract and purified anthocyanin: derivatives of pelargonidin glycoside	Bark of *Ficus benghalensis* Linn.	Alloxan-induced diabetic dogs and rats	Hypoglycemic effect; stimulate insulin secretion	[74,75]
Freeze-dried powder and crude extract preparations	Gamazumi (*Viburnum dilatatum*)	STZ-induced hyperglycemic rats	Decrease plasma glucose level; antioxidant activity	[76]
Freeze dried Jabuticaba peel containing **19** and **27**	Jabuticaba (*Myrciaria spp*.) peel	Rats under high fat diet	Increase glucose and insulin tolerance; reduce serum insulin resistance; increase HDL level	[77]
Anthocyanin fraction from aqueous methanol (70%) extract **28**	Maqui berry (*Aristotelia chilensis*)	Hyperglycemic obese mice fed high fat diet	Improve fasting blood glucose level and glucose tolerance	[39]
Aqueous ethanol (50%) extract containing **1**, **19** and **21**	*Morus alba* fruits	Zucker diabetic fatty rats	Decrease glucose level; maintain insulin level and β cell histology	[78]
Juice predominantly containg **19** and **21**	*Morus australis* fruits	Obese and insulin resistance mice fed with high fat diet	Suppress weight gain and insulin resistance; attenuate lipid accumulation; lower the size of adipocytes	[64]
Aqueous ethanol (70%) and the ethyl acetate fraction	Mulberry *(Morus alba* L.) fruit	STZ induced diabetic mice	Hypoglycemic effect	[79]
Aqueous extract	Mulberry *(Morus alba* L.) fruits	Male Syrian golden hamster	Prevent obesity; reduce hepatic lipogenesis, body weight gain and fat accumulation	[80]
Acidified ethanol extract containing **19** and **20**	Mulberry (*Morus alba* L.) fruit	db/db diabetic mice	β-cell protection; reverse insulin resistance	[41]
Aqueous ethanol (70%) extract	*Mulberry* root bark	STZ induced diabetic rats	Reduces serum glucose and lipid peroxides; increased insulin level	[81]
Concentrated juice	Plums	Wistar fatty rats	Reduce blood glucose; increase insulin sensitivity	[82]
Methanol extract	Pomegranate (*Punica granatum* Linn.) flowers	Zucker diabetic fatty rats	Decreases plasma glucose level	[83,84]
Powder rich in **19**	Purple corn	High fat diet induced insulin resistance mice	Reverse insulin resistance; suppress weight gain and hypertrophy of adipocytes	[85]
Purified anthocyanin: **19**	Purple corn	Diabetic KKA-γ mice	Reduces blood glucose level; enhance insulin sensitivity	[86]
Purified anthocyanin: **34**	Purple sweet potato	Male Sprague Dawley rats	Increase plasma insulin sensitivity; decrease α-glucosidase activity	[87]
Aqueous alcohol extracts containing **25**	Rutgers Scarlet Lettuce	High fat diet induced obese mice	Improve glucose metabolism; decrease total liver lipid	[40]
Aqueos alcohol extract	*Sapindus mukorossi* fruits	STZ induced diabetic rats	Decrease blood glucose level and lipid level	[88]
Aqueous and ethyl acetate extracts	Serviceberry plant samples (leaves, twig and berries)	Diet induced obese and hyperglycemic mice	Lower blood glucose; delay absorption of carbohydrate; inhibit intestinal α-glucosidase activity	[35]
Purified anthocyanin: **20**, **21** and **31**	Sweet cherry *(Prunus avium)*	Male mice fed with high fat diet	Prevent body weight gain; reduces size of adipocytes; decrease leptin secretion, serum glucose triglyceride, total cholesterol and low density lipoprotein	[89]
Anthocyanin-enriched juices and purified **19**	Sweet orange (*Citrus sinensis*), Moro (a blood orange)	Obese mice fed with high fat diet	Improve glucose tolerance, insulin sensitivity; reduce hepatic accumulation of lipid	[90]
Freeze-dried	Tart cherries	Male rats	Decrease fasting glucose level; increase plasma insulin level	[91]

* The full list of compounds designated with bold numbers is presented in Table 1.

**Table 4:** Current clinical trials on anthocyanins as potential therapy against insulin resistance and/or diabetes/obesity.

Clinical Trial Identifier No.	Objective	Voluntary and Dose	Status
NCT01245270	Single supplement of standardized bilberry extract (36% *w*/*w* anthocyanins) modifies glycemic response in persons with type-2 diabetes controlled by diet and lifestyle	8 male patients of age between 40 and 70 years with type-2 diabetes given a single oral capsule of 0.47 g standardized blueberry extract followed by a polysaccharide drink in a double blind cross over intervention	Completed
NCT01005420	The effect of anthocyanins in the form of blueberry powder on enhancing insulin sensitivity in insulin resistant and obese human	37 male and female of age 20 years and older taking 45 g of blueberry powder per day as a smoothie	Completed
NCT02689765	Effect of purified anthocyanins from bilberries and black currant on insulin resistance, glucose and lipid metabolism disorders	160 humans both male and female with type-2 diabetes of age 40–75 years taking two 80 mg anthocyanin capsule twice a day for 24 weeks	Completed
NCT 01883401	Investigate the effect of low and high doses of freeze-dried strawberries in cardiovascular risk factor in subjects with abnormal adiposity and dyslipidemia	60 male and female patients of age between 19 and 72 years were given 25 and 50 g of dried strawberries	Completed
NCT 02340039	The acute effect of black currant and apple extract on postprandial glycemia	34 male and female patients of age between 20 and 60 years were given 600 mg of black current anthocyanins and 600 mg of apple polyphenols	Completed
NCT 02650726	Effect of purified anthocyanins on high density lipoprotein and endothelial function in subjects with type-2 diabetes	80 male and female patients of age between 40 and 60 years were given daily dose of 320 mg anthocyanin for 24 weeks in a randomized double blinded placebo-controlled trial	Completed
NCT 01053793	A trial to measure the glycemic index and polyphenol bioavailability of four different varieties of potato	10 male and female patients of age between 18 and 50 years were given 50 g of cooked purple, red, yellow and white potatoes	Completed
NCT 02317211	Effect of purified anthocyanins on oxidative stress and glycemic control in subjects with type-2 diabetes	70 patients of age between 25 and 65 years were given 320 mg anthocyanin daily for 12 weeks in a randomized double blinded placebo-controlled trial	Completed
NCT 01720511	Pilot study of the effect of purple rice on glucose tolerance, serum lipid and inflammation	10 male and female patients of age 18 years and above were given one cup of rice into their dishes and consumed at lunch and dinner each day for 4 weeks (equivalent to 4 ounces of uncooked rice/day)	Completed
NCT O2035592	Dose dependent impact of blueberry powder intake on insulin sensitivity and resistance	144 male and female of age 50–74 years	Active
NCT 02291250	Effect of black currant containing anthocyanins on glucose metabolism	16 obese male female of 21–70 age	Recruiting
NCT02972996	Effect of blueberry consumption on cardiometabolic prevention in type-2 diabetes patients	48 male patients of age between 45 and 75 years were given 22 g of blueberry powder in a randomized experiment	Recruiting
NCT 03213288	The effect of bilberry fruit and black rice derived anthocyanins on lipid status in adults	50 male and female patients of age 45 years and older were given 320 mg of anthocyanins for 28 days	Recruiting
NCT 02940080	Effect of anthocyanins extracted from purple potatoes on healthy study subjects Postprandial glycaemia and insulinemia	20 male patients of age between 18 and 45 years were given 168 mg of anthocyanins extracted from purple-flashed potatoes added to steam-cooked mashed potatoes in water	Recruiting
NCT 02291250	Effect of soft fruit on postprandial blood glucose	16 overweight male and female patients of age between 21 and 70 years were given black current (200 g) and green currant (200 g)	Recruiting
NCT01180712	Study of oral anthocyanins on insulin resistance	60 obese type-2 diabetic males of age 40–70 years taking 1.4 g of concentrate blueberry extract in a hard gelatin capsule administered thrice a day for 21 days	Recruiting

(NCT numbers refer to the source of www.clinicalTrails.gov).
